# Genetic diversity and natural selection on the thrombospondin-related adhesive protein (TRAP) gene of *Plasmodium falciparum* on Bioko Island, Equatorial Guinea and global comparative analysis

**DOI:** 10.1186/s12936-021-03664-8

**Published:** 2021-03-02

**Authors:** Li-Yun Lin, Hui-Ying Huang, Xue-Yan Liang, Dong-De Xie, Jiang-Tao Chen, Hua-Gui Wei, Wei-Yi Huang, Carlos Salas Ehapo, Urbano Monsuy Eyi, Jian Li, Jun-Li Wang, Yu-Zhong Zheng, Guang-Cai Zha, Yu-Ling Wang, Wei-Zhong Chen, Xiang-Zhi Liu, Huan-Tong Mo, Xin-Yao Chen, Min Lin

**Affiliations:** 1 School of Food Engineering and Biotechnology, Hanshan Normal University, Chaozhou, Guangdong People’s Republic of China; 2 Department of Medical Laboratory, Chaozhou People’s Hospital Affiliated to Shantou University Medical College, Chaozhou, Guangdong People’s Republic of China; 3 Shantou University Medical College, Shantou, Guangdong People’s Republic of China; 4 Department of Medical Laboratory, Huizhou Central Hospital, Huizhou, Guangdong People’s Republic of China; 5 Department of Medical Laboratory, Foshan Second People’s Hospital, Foshan, Guangdong People’s Republic of China; 6 The Chinese Medical Aid Team To the Republic of Equatorial Guinea, Guangzhou, Guangdong People’s Republic of China; 7 School of Clinical Medicine, Youjiang Medical University for Nationalities, Baise, China; 8 Department of Medical Laboratory, Malabo Regional Hospital, Malabo, Equatorial Guinea; 9 Department of Human Parasitology, School of Basic Medical Sciences, Renmin Hospital, Hubei University of Medicine, Shiyan, Hubei People’s Republic of China; 10 Department of Infectious Diseases, Renmin Hospital, Hubei University of Medicine, Shiyan, Hubei People’s Republic of China

**Keywords:** *Plasmodium falciparum* thrombospondin-related adhesive protein (*Pf*TRAP), Genetic diversity, Natural selection, Bioko Island, Malaria, Vaccine candidate

## Abstract

**Background:**

Thrombospondin-related adhesive protein (TRAP) is a transmembrane protein that plays a crucial role during the invasion of *Plasmodium falciparum* into liver cells. As a potential malaria vaccine candidate, the genetic diversity and natural selection of *Pf*TRAP was assessed and the global *Pf*TRAP polymorphism pattern was described.

**Methods:**

153 blood spot samples from Bioko malaria patients were collected during 2016–2018 and the target TRAP gene was amplified. Together with the sequences from database, nucleotide diversity and natural selection analysis, and the structural prediction were preformed using bioinformatical tools.

**Results:**

A total of 119 Bioko *Pf*TRAP sequences were amplified successfully. On Bioko Island, *Pf*TRAP shows its high degree of genetic diversity and heterogeneity, with π value for 0.01046 and Hd for 0.99. The value of dN–dS (6.2231, p < 0.05) hinted at natural selection of *Pf*TRAP on Bioko Island. Globally, the African *Pf*TRAPs showed more diverse than the Asian ones, and significant genetic differentiation was discovered by the fixation index between African and Asian countries (Fst > 0.15, p < 0.05). 667 Asian isolates clustered in 136 haplotypes and 739 African isolates clustered in 528 haplotypes by network analysis. The mutations I116T, L221I, Y128F, G228V and P299S were predicted as probably damaging by PolyPhen online service, while mutations L49V, R285G, R285S, P299S and K421N would lead to a significant increase of free energy difference (ΔΔG > 1) indicated a destabilization of protein structure.

**Conclusions:**

Evidences in the present investigation supported that *Pf*TRAP gene from Bioko Island and other malaria endemic countries is highly polymorphic (especially at T cell epitopes), which provided the genetic information background for developing an *Pf*TRAP-based universal effective vaccine. Moreover, some mutations have been shown to be detrimental to the protein structure or function and deserve further study and continuous monitoring.

**Supplementary Information:**

The online version contains supplementary material available at 10.1186/s12936-021-03664-8.

## Background

Malaria is a major public health threat in many parts of the globe and is responsible for half a million deaths annually. According to the World Malaria Report 2019, an estimated 228 million persons suffered from malaria worldwide, with 435,000 malaria deaths in 2018 [1]. *Plasmodium falciparum* is the parasite that cause 99.7% malaria cases in African regions [1].

The sub-Saharan African country, Equatorial Guinea has a total population of 1.31 million (2018). Bioko, an island of Equatorial Guinea off the coast of Cameroon, with historically high malaria transmission. The Bioko Island Malaria Elimination Project (BIMEP) is a fusion of two long-standing anti-malaria programmes (Bioko Island Malaria Control Project, BIMCP and Equatorial Guinea Malaria Vaccine Initiative, EGMVI) in Equatorial Guinea, with Medical Care Development Institution (MCDI) as the lead implementing partner [2–4]. Recently evaluations indicated that malaria prevalence had dropped considerably from 43.3 to 10.5% between 2004 and 2016, resulting in a 13.3% reduction of moderate to severe anaemia in children aged 1–5 years. Despite considerable success in reducing the burden on the island, malaria is still a major public health concern in Bioko Island.

Malaria infection in humans starts when infected female *Anopheles* mosquitoes take their blood meal and inject sporozoites into the host (human) skin. Sporozoites quickly pass into the liver, where they infect hepatocytes. Thrombospondin-related adhesive protein (TRAP) is the most extensively studied *Plasmodium* transmembrane protein and it would be released on the surface when the sporozoite into contact with the host cell [5]. *Plasmodium* TRAP also assists the sporozoite in several pivotal functions, such as sporozoite gliding motility, hepatocyte invasion and establishment of infection in the vertebrate host [6–8]. The *P. falciparum* TRAP (*Pf*TRAP) extracellular domain (ECD) consists of three domains/motifs that include the A-domain (similar to the A-or I-domain which is found in integrins), the TSP (thrombospondin repeat motif, a heparin-binding module, also called the RII region) and a proline-rich segment at the C-terminus [9]. The previous studies [10] revealed a higher frequency of nonsynonymous to synonymous single nucleotide polymorphisms (SNPs) of TRAP within the *P. falciparum* population of Gambian and Thailand. In their studies, McDonald–Kreitman test showed that the ratio of the number of nonsynonymous to synonymous SNPs within *P. falciparum* was significantly higher than that of the number of nonsynonymous to synonymous fixed sites between *P. falciparum* and *Plasmodium reichenowi*. Furthermore, the value of Tajima’D test and Fu and Li’s test also suggested that the TRAP gene is under diversifying selection in the *P. falciparum* population in Gambian and Thailand [10]. However, no evidence for balancing selection was reported for parasites obtained from sub-Saharan African countries, except The Gambia. Thus, it remains unclear whether positive selection acts on the TRAP gene of *P. falciparum* in other geographic regions of sub-Saharan Africa.

The aims of the present study were to investigate whether the TRAP gene is under diversifying selection in Bioko *P. falciparum* and to elucidate how TRAP gene is differentiated among *P. falciparum* populations. This research would be helpful not only for understanding the molecular evolution of the TRAP gene in *P. falciparum* but also for the improvement of peptide vaccines based on the TRAP antigen.

## Methods

### Study area

This study was carried out in Malabo Regional Hospital, in private diagnostic clinics (PDCs) in different regions of Bioko Island, and in the clinic of the Chinese medical aid team to the Republic of Equatorial Guinea. Bioko is an island 32 km off the west coast of Africa, and the northernmost part of Equatorial Guinea. The island has a population of 334,463 inhabitants (2015 census, of which approximately 90% live in Malabo, the capital city of Equatorial Guinea) and a humid tropical environment. The launch of the BIMCP has had a marked impact on malaria transmission, with the decrease of parasite prevalence from over 45% in 2004 to 8.5% in 2016 and the reduction of entomological inoculation rate (EIR) from more than 1000 before 2004 to 14 in 2015 (www.mcdinternational.org) in Bioko Island.

### Samples collection

A total of 153 blood spot samples were collected from the patients with uncomplicated malaria during January 2016–December 2018. Consents were obtained from all participating subjects or their parents and the ethical approval was obtained from the Ethics Committee of Malabo Regional Hospital. Included patients were aged between 4 months and 80 years, were residents on Bioko Island. Malaria patients were classified into uncomplicated malaria states according to World Health Organization (WHO) criteria, which were defined as positive smear for *P. falciparum* and presence of fever (≥ 37.5 °C). Dried blood spots were collected on day zero of enrollment through finger prick bleeding spotted onto Whatman 903® filter paper (GE Healthcare, Pittsburgh, USA) for future use. Laboratory screening for malaria was done using rapid diagnostic test (RDT) and confirmed using microscopic examination of blood smears. For quality control, archived malaria-positive microslides were re-examined, and parasite density was recorded; The *Plasmodium* species were identified by a real-time PCR followed by high-resolution melting (HRM) [11]. The pGEM-T standard plasmids of the four human *Plasmodium* species, *P. falciparum*, *Plasmodium ovale*, *Plasmodium malariae* and *Plasmodium vivax* were used as control. In this study, although only clear non-superimposed signals of the sequences were included from electropherograms for further analysis, these sequences could derive from either monoclonal infections or the most predominant clones in these isolates.

### Genomic DNA extraction

Parasite genomic DNA was extracted from dried filter blood spots by Chelex-100 extraction method described in a previous article [12]. The DNA products were collected in sterile tubes and stored at − 80 °C in reserve.

### Sequencing analysis of the entire TRAP gene

The full-length TRAP gene (NCBI Gene ID: 814170) was divided into two segments and amplified by nested PCR. The primers designed for nested PCR are presented in Table 1. For the first round PCR, l μl of genomic DNA was amplified with 12.5 μl 2 × Master Mix (DNA Polymerase, dNTP Mixture, PCR buffer), 1 μl 10 nM outer forward primer, 1 μl 10 nM outer reverse primer, and sterile ultra-pure water to a final volume of 25 μl. Thermal cycling parameters for PCR were as follows: initial denaturation at 94 °C for 3 min; 30 cycles of 94 °C for 30 s, annealing at 55 °C for 30 s and extension at 72 °C for 2 min; followed by a final extension step at 72 °C for 5 min. For the second round PCR, 2 μl of the primary PCR product was amplified in a 50 μl reaction volume comprised of 25 μl 2 × Master Mix (DNA Polymerase, dNTP Mixture, PCR buffer), 2 μl 10 nM inner forward primer, 2 μl 10 nM inner reverse primer, and sterile ultrapure water to a final volume of 50 μl. All PCR products were analysed using 1.2% agarose gel electrophoresis, and then, they were purified and sequenced by using an ABI 3730 × L automated sequencer (Shanghai Yingjun Biotechnology Co., LTD, Guangzhou branch). To ensure the accuracy of the sequencing, each isolate was sequenced at least twice. Sequencing primers were the reverse primers of the second round PCR. All the sequences were analysed and integrated by MEGA6 software [13]. These nucleotide sequences have been deposited at NCBI under Accession Numbers (MK981410–MK981530).

**Table 1:** Primer design information of *Pf*TRAP gene

Primer name	Primer sequence	Production length (bp)	Start position	End position
TRAP-1-outter-F	5′-GATATCACACCAAATAAATTACAC-3′	1398	− 152	1247
TRAP-1-outter-R	5′-GATTATCGTGCTTATTTTCGG-3′			
TRAP-1-inner-F	5′-GTATGTGCATGCGTACAAG-3′	1288	− 82	1207
TRAP-1-inner-R	5′-GTTTTCTTCTCGATCGTCT-3′			
TRAP-2-outter-F	5′-CACTAAATCCAGAAGAAGGAA-3′	902	986	+ 162
TRAP-2-outter-R	5′-CAGCTTATTCTTTTTTATCCTTAC-3′			
TRAP-2-inner-F	5′-GAAAATCCAGAAAATCCACC-3′	727	1039	+ 50
TRAP-2-inner-R	5′-GTTGTTGTGTATTTCACTATATTAC-3′			

The Start position number with minus sign (−) indicated the number of nucleotides that before TRAP 5′ terminus. The End position number with plus sign (+) indicated the number of nucleotides that after TRAP 3′ terminus

### Sequence polymorphism and natural selection analysis

The nucleotide and deduced amino acid sequences of *Pf*TRAP were analysed using EditSeq and SeqMan in the DNASTAR package (DNASTAR, Madison, WI, USA). The *Pf*TRAP sequence of the laboratory-adapted *P. falciparum* strain 3D7 (NC_004331.3) was included in the alignment for comparison as a reference sequence. The values of segregating sites (S), the number of haplotypes (H), haplotype diversity (Hd), and nucleotide diversity (π) were calculated using DnaSP version 5.10.00 [14]. In order to test the null hypothesis of neutrality of *Pf*TRAP, the rates of synonymous (dS) and nonsynonymous (dN) substitutions were estimated and were compared using the Z-test (P < 0.05) in MEGA6 program [13] using Nei and Gojobori’s method [15] with the Jukes and Cantor (JC) correction of 1000 bootstrap replications. Tajima’s D test [16] were performed using DnaSP ver. 5.10.00 in order to evaluate the neutral theory of natural selection. The probability of recombination between adjacent sites per generation (Rb), and the minimum number of recombination events (Rm) were analysed using DnaSP ver. 5.10.00.

### Global PfTRAP sequences acquisition and comparative analysis

In order to analyse the genetic diversities and natural selection of *Pf*TRAP among global *P. falciparum* isolates, a total of 1287 sequences from 13 malaria endemic regions (Bangladesh, Cambodia, Congo, Gambia, Ghana, Guinea, Laos, Malawi, Mali, Myanmar, Senegal, Thailand and Vietnam) were acquired by mining the MalariaGEN *Pf*3k Project database (release 5) [16, 17] using samtools [18] and vcftools [19]. Genetic polymorphism and tests of neutrality were calculated for each population using DnaSP ver. 5.10.00 and MEGA6 as described above. In order to investigate the genetic relationships among global *Pf*TRAP haplotypes, the haplotype network for 1406 full-length sequences of *Pf*TRAP from Bioko and other countries listed above was constructed by Network 10.1.0.0 program using Median-Joining method [20].

### Prediction of impact of amino acid change upon protein structure

The 3-dimensional structure of *Pf*TRAP (3D7 isolate) was predicted and modeled by I-TASSER online server (https://zhanglab.ccmb.med.umich.edu/I-TASSER/) [21] and presented using YASARA software [22]. PolyPhen-2 [23] online serve was used to predict potential impact of amino acid substitutions on the structure or function. A prediction of probably damaging indicates that the query substitution is predicted to be damaging with high confidence, while a prediction of benign indicates that the query substitution is predicted to be benign with high confidence. A prediction of possibly damaging indicates that the query substitution is predicted to be damaging, but with low confidence. Using FOLDX plugin [24] in YASARA [22] to predict the changes in free energy before and after the mutations: ΔΔG(change) = ΔG(mutation)  − ΔG(wild-type). As a rule of thumb for use: ΔΔG (change) > 0: the mutation is destabilizing; ΔΔG (change) < 0: the mutation is stabilizing.

## Results

### Genetic polymorphism and natural selection of PfTRAP on Bioko Island

Of the 153 blood samples extracted from Bioko Island, 121 yielded suitable *Pf*TRAP amplicons for sequencing and deposited to NCBI Genbank (MK981410–MK981530). Finally, 119 monoclonal sequences were applied in the further analysis while 2 polyclonal sequences (MK981439, MK981530) were excluded.

As the result of genetic polymorphism and natural selection analysis shown in Table 2, on Bioko Island, a total of 87 haplotypes were found among 119 samples, with the haplotype diversity (Hd) for 0.99. Furthermore, nucleotide diversity (π) of Bioko Island was detected as 0.01046, which was a relatively high value among the 14 countries in this analysis. As for the parameters related to natural selection, the value of dN–dS was 6.2231 (p < 0.05), which hinted at natural selection on Bioko Island. The value of Tajima’s D of Bioko Island was detected as − 0.41438, which was not significant based on Tajima’s D significance levels [16]. To analyse the recombination degree of *Pf*TRAP on Bioko Island, the minimum number of recombination event (Rm) and the probability of recombination between adjacent sites (Rb) was detected as 22 and 119, respectively, which shows relatively high level among the 14 countries or areas included in this analysis.

**Table 2:** Result of genetic diversity, natural selection and recombination among global *Pf*TRAP sequences

Countries	n	S	H	Hd	π	Tajima’s D	dN-dS	Rm	Rb
Africa									
Bioko	119	100	87	0.99	0.01046	− 0.41438	6.2231*	22	119
Congo	54	76	51	0.997	0.01029	− 0.08216	6.3986*	19	298
Gambia	34	65	28	0.985	0.01025	0.04683	6.9506*	16	372
Ghana	228	87	193	0.9968	0.01075	0.47289	7.0314*	27	287
Guinea	49	73	43	0.993	0.009778	− 0.19876	6.6430*	21	162
Malawi	106	75	84	0.993	0.01066	0.61245	6.0216*	26	124
Mali	39	67	39	0.999	0.01043	0.12807	7.4272*	16	339
Senegal	110	76	64	0.977	0.01006	0.32976	7.2140*	19	142
Asia									
Thailand	104	40	29	0.947	0.00546	0.61617	4.6708*	14	62.4
Vietnam	60	40	26	0.957	0.00567	0.37851	5.0222*	12	45.2
Myanmar	45	40	22	0.954	0.00507	− 0.22333	4.8873*	10	40.4
Bangladesh	23	53	23	1	0.00769	− 0.34671	5.8936*	14	53.7
Cambodia	393	47	71	0.924	0.00517	0.52199	4.8661*	18	35
Laos	42	41	25	0.961	0.00614	0.21991	5.2813*	11	52

n for number of sequences; S for segregating sites; H number of haplotypes; Hd for haplotype diversity; π for nucleotide diversity; dN for number of non-synonymous substitutions per non-synonymous site; dS for number of synonymous substitutions per synonymous site; Rm for minimum number of recombination event; Rb for the probability of recombination between adjacent sites; * for *p* < 0.05

### Global PfTRAP comparative analysis

Not only Bioko Island, but also 7 other African countries and 6 Asian countries were included in the genetic diversity and natural selection analysis of *Pf*TRAP. 1287 global sequences mined from *Pf*3K database (23 for Bangladesh, 393 for Cambodia, 54 for Congo, 34 for Gambia, 228 for Ghana, 49 for Guinea, 42 for Laos, 106 for Malawi, 39 for Mali, 45 for Myanmar, 110 for Senegal, 104 for Thailand, 60 for Vietnam, shown in Additional file 4) and 119 Bioko sequences were applied in the global comparative analysis. According to the results, a total of 161 SNPs were detected from the 1406 *Pf*TRAP sequences (7 SNPs for DomainI, 41 for DomainII, 23 for DomainIII, 83 for DomainIV, 3 for DomainV and 4 for DomainVI, respectively), among them there were 42 SNPs appeared only once. Due to the uncertainty, these single SNPs were not included in the following analysis (original SNP information was shown in Additional file 1). There were 7 SNPs (S91N, K130R, S179N, L287P, N317K, S419P and A489G) popular globally, with percentage over 80%, and notably, there are 60 SNPs unique to Africa, while there were 12 SNPs unique to Asia. Not surprisingly, no mutations were found in some important motifs including WSPCSVTCG in A-domain, metal ion-dependent adhesion site (MIDAS), IQQ motif, A glycoprotein with a cellular recognition function (RGD) and two important points (aa131 and aa162).

**Fig. 1:**
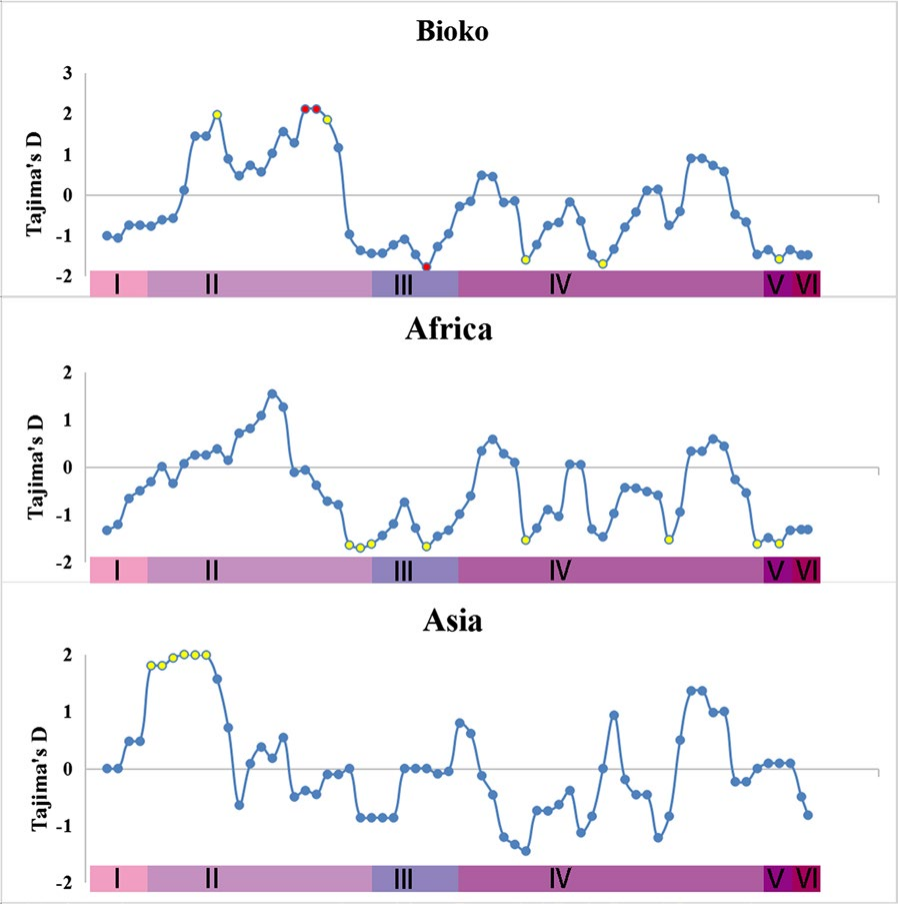
Trend chart of Tajima’s D value of *Pf*TRAP from Africa, Asia and Bioko Island. Red dot indicated *p* value < 0.05; Yellow dot indicated *p* value < 0.1; Blue dot indicated *p* value > 0.1. DomainI to Domain VI were presented in different color

As shown in Table 2, both Hd and π show that the polymorphism in Africa is greater than in Asia (except Bangladesh). All the 14 countries and areas in this analysis shown evidences of natural selection with the positive value of dN–dS (p < 0.05). Except for Bioko, Congo, Guinea, Myanmar, Bangladesh, the Tajima’s D of other 9 countries shown positive value, which signified low levels of both low and high frequency polymorphisms, indicating a decrease in population size and/or balancing selection (Table 2). In the sight of the Tajima’s D of the full-length *Pf*TRAP, on Bioko, the value of DomainII is positive and obviously deviated from 0, while most of the rest region got the negative value (Fig. 1). The overall trend is similar in Bioko and Africa, while in Asia, higher positive Tajima’s D was found in N-terminal of DomainII and C-terminal of DomainIV (Fig. 1). When turns to the recombination effect, the parameters (Rm and Rb) revealed that more frequent recombination events were taking place in Africa rather than Asia (Table 2).

**Fig. 2:**
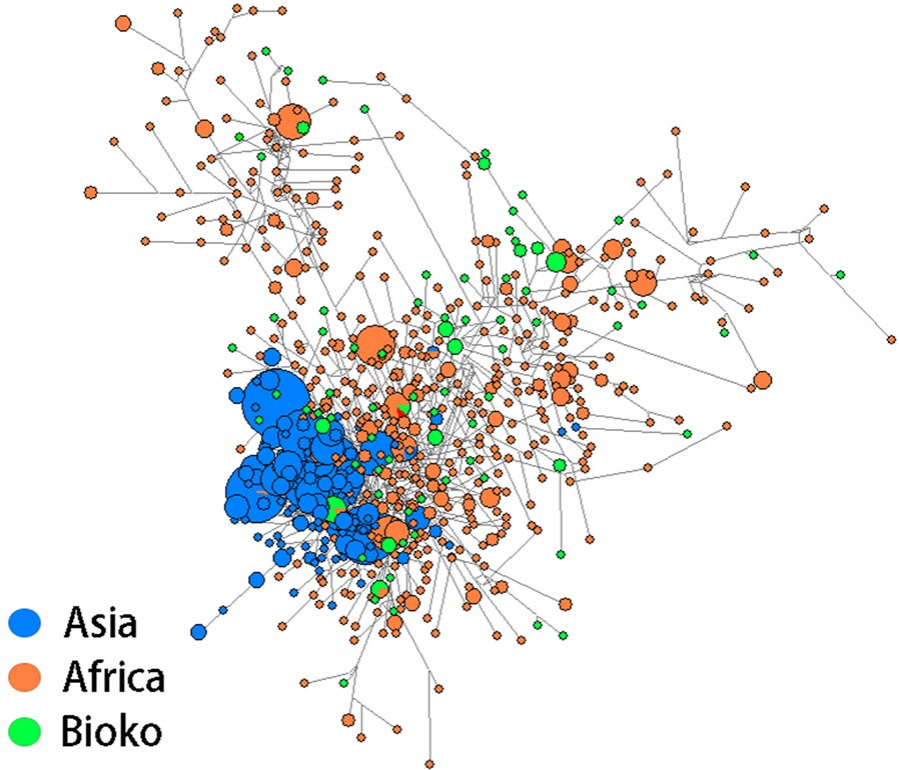
Haplotype network of global *Pf*TRAP sequences. Each circle stands for one haplotype. The size of circle indicated the sample size of the haplotype. The length of the line connecting two haplotypes indicated the genetic distance. Blue circles represent sequences from Asia; Orange circles represent sequences from Africa; Green circles represent sequences from Bioko Island

For further exploration of the relationship of haplotypes, a haplotype network was constructed based on global *Pf*TRAP sequences (excluded repeat region) using NETWORK software. A total of 661 haplotypes were detected among 1406 isolates (Fig. 2), among them, 667 Asian isolates clustered in 136 haplotypes and 739 African isolates clustered in 528 haplotypes, which indicated the higher heterogeneity found among African *P. falciparum* population. Generally, the vast majority of haplotypes were limited to one continent (Africa or Asia) and only several haplotypes (Hap_8, Hap_18, Hap_69, Hap_104 and Hap_370) were shared between two continents isolates (Detailed information about haplotypes was shown in Additional files 2, 3 and 5). In the meantime, it was found that the haplotypes of Bioko Island isolates were scattered. Obviously, haplotypes from Asian isolates were distributed relatively concentrated while the haplotypes from African isolates were in greater dispersion with long branch, which indicated that the recent mutations have been more active in Africa than Asia. In order to analyse the genetic differentiation among *Pf*TRAPs from 14 malaria endemic regions, Fixation Index (FST) was calculated and shown in Table 3. According to Sewall Wright rules, FST range from 0 to 0.05 reflects little genetic differentiation; FST range from 0.05 to 0.15 means moderate genetic differentiation; FST range from 0.15 to 0.25 for great genetic differentiation, while FST over 0.25 means extremely high genetic differentiation. According to Table 3, it seems like the TRAP gene from Bioko Island have not so much genetic differentiation with other 7 African countries in this analysis according to the FST between them shows little or moderate level of genetic differentiation (p < 0.05). The FST between Bioko and Asian countries were over 0.15 (*p* < 0.05), which indicated a greater genetic differentiation. Generally, the differentiation of *Pf*TRAP among African countries is less pronounced, and the same phenomenon was found among Asian countries, but the high degree of genetic differentiation between African and Asian countries cannot be ignored (*p* < 0.05).

**Table 3:** Result of Fixation Index (FST) of *Pf*TRAP between populations of 14 countries and 3D7 isolate

	3D7	Bioko	Congo	Gambia	Ghana	Guinea	Malawi	Mali	Senegal	Thailand	Vietnam	Myanmar	Bangladesh	Cambodia
Bioko	0.01884													
Congo	0.04111	**0.03557**												
Gambia	− 0.02608	**0.04804**	**0.02839**											
Ghana	− 0.00027	**0.04754**	**0.031**	0.00462										
Guinea	− 0.01109	**0.05875**	**0.03512**	− 0.01171	**0.0105**									
Malawi	0.04597	**0.04828**	**0.0117**	**0.04733**	**0.03895**	**0.05173**								
Mali	0.02442	**0.08476**	**0.06783**	0.00006	**0.01863**	0.00512	**0.07585**							
Senegal	0.01457	**0.06236**	**0.04189**	− 0.0032	**0.00733**	0.00253	**0.05327**	0.00427						
Thailand	0.41365	**0.18493**	**0.2088**	**0.1872**	**0.17375**	**0.18402**	**0.20101**	**0.20032**	**0.19828**					
Vietnam	**0.39716**	**0.1835**	**0.20929**	**0.17677**	**0.17109**	**0.17222**	**0.19248**	**0.18318**	**0.18338**	0.01441				
Myanmar	0.45549	**0.20845**	**0.22712**	**0.20579**	**0.19298**	**0.20113**	**0.2189**	**0.2141**	**0.20958**	− 0.00322	0.01804			
Bangladesh	0.32227	**0.1773**	**0.18833**	**0.15792**	**0.15682**	**0.15563**	**0.18121**	**0.17234**	**0.16962**	**0.03613**	0.01636	0.45549		
Cambodia	**0.37617**	**0.19574**	**0.21869**	**0.18226**	**0.17058**	**0.17901**	**0.20142**	**0.18779**	**0.18695**	**0.02722**	− 0.01528	**0.0316**	**0.0352**	
Laos	**0.37235**	**0.17651**	**0.19926**	**0.16232**	**0.15973**	**0.15977**	**0.18318**	**0.16318**	**0.16903**	**0.0392**	− 0.02372	**0.03975**	0.01368	− 0.01303

The value in bold is statistically significant (*p* < 0.05)

### Effect prediction of mutations located at immune epitopes

**Fig. 3:**
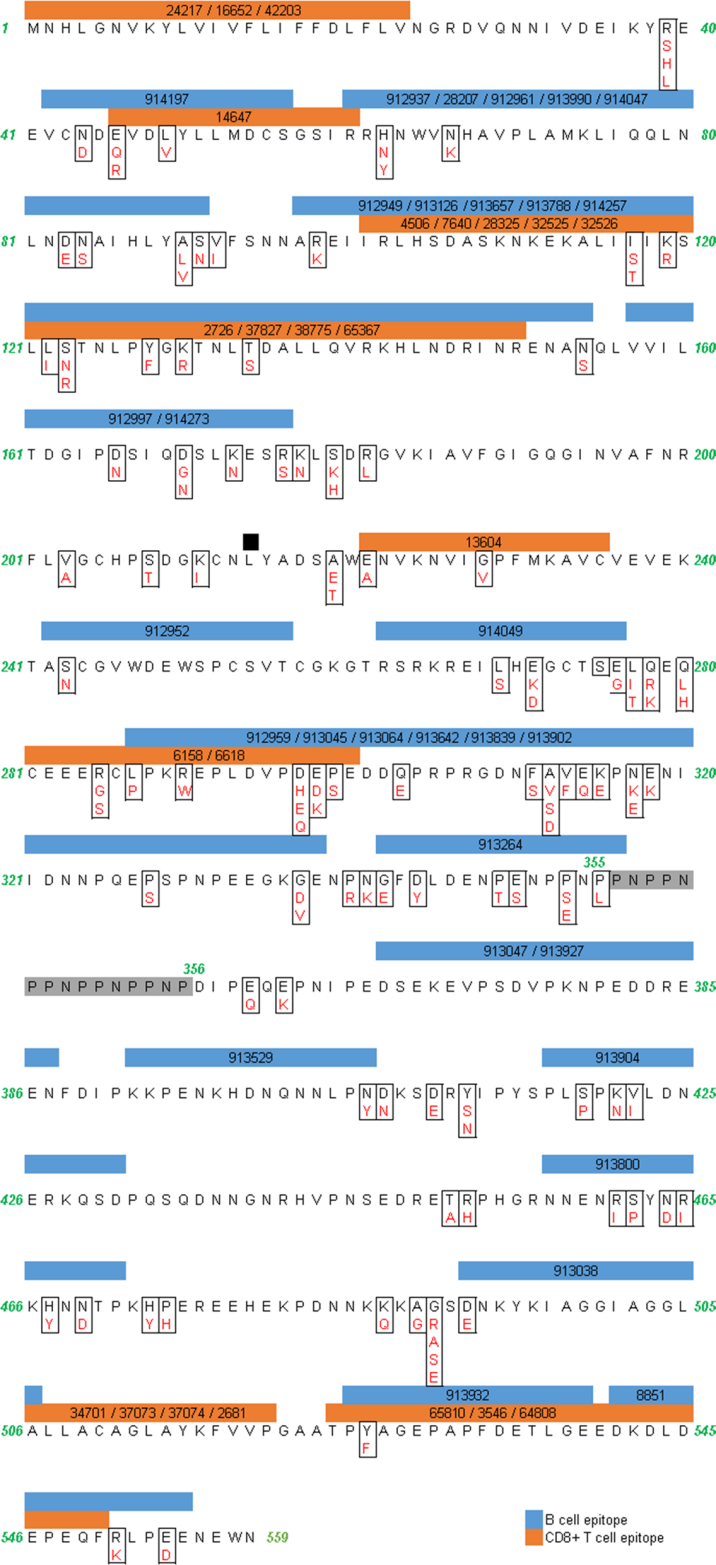
CD8+ T cell and linear B cell Epitopes and amino acids substitution distribution of *Pf*TRAP gene. The full-length TRAP sequences (1–559aa) was referred to the 3D7 isolates (NCBI Gene ID: 814170). Sequences with blue block above is linear B cell epitope; Sequences with orange block above is CD8+ T cell epitope (Detail information of CD8+ T cell epitopes was shown in Additional File 6); Numbers in the color blocks are the corresponding epitope ID of IEDB database. Sequences in gray block is the repeat region. Amino acid in red is for mutant

In this study, all the proven T cell epitopes, B cell epitopes, as well as nonsynonymous substitutions were marked and presented in Fig. 3. As it shown, 98 amino acid substitutions were located at the B cell epitopes while 25 substitutions were at T cell epitopes. 24 substitutions located at the overlap region of T cell epitope and B cell epitope. Since previous reports shown that cellular immunity is more dominant than humoral immunity in the *Pf*TRAP-based vaccine clinical trials, the following mutation effect prediction analysis was focus on the variations at T cell epitopes. In Table 4, among the 30 mutations in T cell epitopes, 13 of them were predicted as benign and 12 for possibly damaging. Not surprisingly, the high frequency (> 90%) mutations were all predicted as benign. It is worth noting that there were 5 mutations (I116T, L221I, Y128F, G228V and P299S) predicted as probably damaging and interestingly, all these 5 mutations were mostly or totally distributed in Africa region, with the occurrence frequency ranging from 0 to 28% worldwide.

**Table 4:** Information of mutations at T cell epitopes and mutation effect prediction

Region	Mutations	Frequency in global	Frequency in Bioko	Distribution (Africa%/Asia%)	Polyphen score	ΔΔG
A-Domain	E 46Q	24%	32%	75/25	0.893*	− 0.211536
	E 46R	0%(7/1406)	0%	100/0	0.732*	0.0221299
	L 49 V	1%	3%	100/0	0.944*	1.12894
	I116S	13%	19%	99/1	0.924*	0.342025
	I116T	0%(2/1406)	0%	100/0	0.965**	− 0.386652
	K119R	13%	16%	52/48	0.020	− 0.194173
	L122I	2%	7%	100/0	0.965**	0.571158
	S123N	19%	7%	57/43	0.000	− 0.26044
	S123R	0%(2/1406)	0%	50/50	0.184	− 0.349001
	Y128F	21%	28%	98/2	0.965**	0.770957
	K130R	93%	94%	49/51	0	0.0703724
	T134S	37%	24%	43/57	0.463*	0.705496
TSR	A219E	2%	0%	8/92	0.122	− 0.166547
	A219T	2%	8%	87/13	0.954*	− 0.345222
	E221A	0%(6/1406)	1%	100/0	0.924*	0.128533
	G228V	3%	3%	100/0	0.997**	0.411189
DIV	R285G	1%	0%	100/0	0.663*	1.3286
	R285S	1%	0%	100/0	0.663*	1.16481
	L287P	97%	95%	51/49	0.000	0.546262
	R290W	29%	61%	72/28	0.001	0.336084
	D297H	54%	29%	47/53	0.000	− 0.336216
	D297E	5%	8%	92/8	0.000	− 0.352555
	D297Q	9%	14%	25/75	0.000	− 0.524996
	E298D	2%	0%	100/0	0.544*	− 0.0333006
	E298K	0%(4/1406)	0%	100/0	0.893*	− 1.10525
	P299S	1%	5%	100/0	0.958**	1.17995
	S419P	96%	90%	51/49	0.000	− 0.8004
	K421N	29%	41%	90/10	0.051	1.41483
	V422I	0%(3/1406)	2%	100/0	0.455*	− 0.147794
TM (DV)	Y526F	4%	0%	0/100	0.051	0.56536

Polyphen score with * indicated possibly damaging mutation; Polyphen score with ** indicated probably damaging mutation. Polyphen score without * indicated benign mutation

**Fig. 4:**
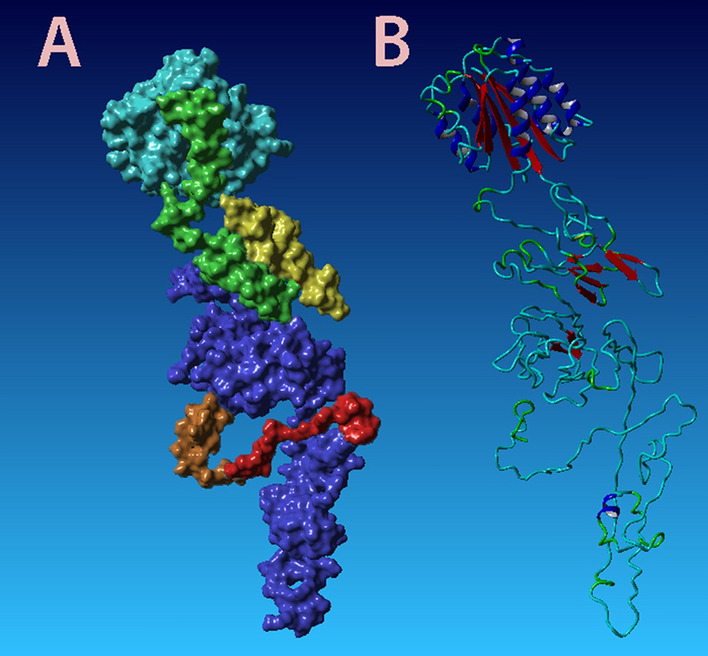
Predicted 3-dimensional structure of TRAP protein. **a** Molecular surface of predicted TRAP structure. Yellow for DomainI (Signal sequences); Cyan for DomainII (A domain); Green for Domain III (TSR region); Blue for Domain IV (Proline-rich region, including repeat region); Red for Domain V (transmembrane domain); Orange for Domain VI (cytoplasmic tail domain). **b** Predicted secondary structure of TRAP protein. Helix, Strand and Coil were shown in different color and form

**Fig. 5:**
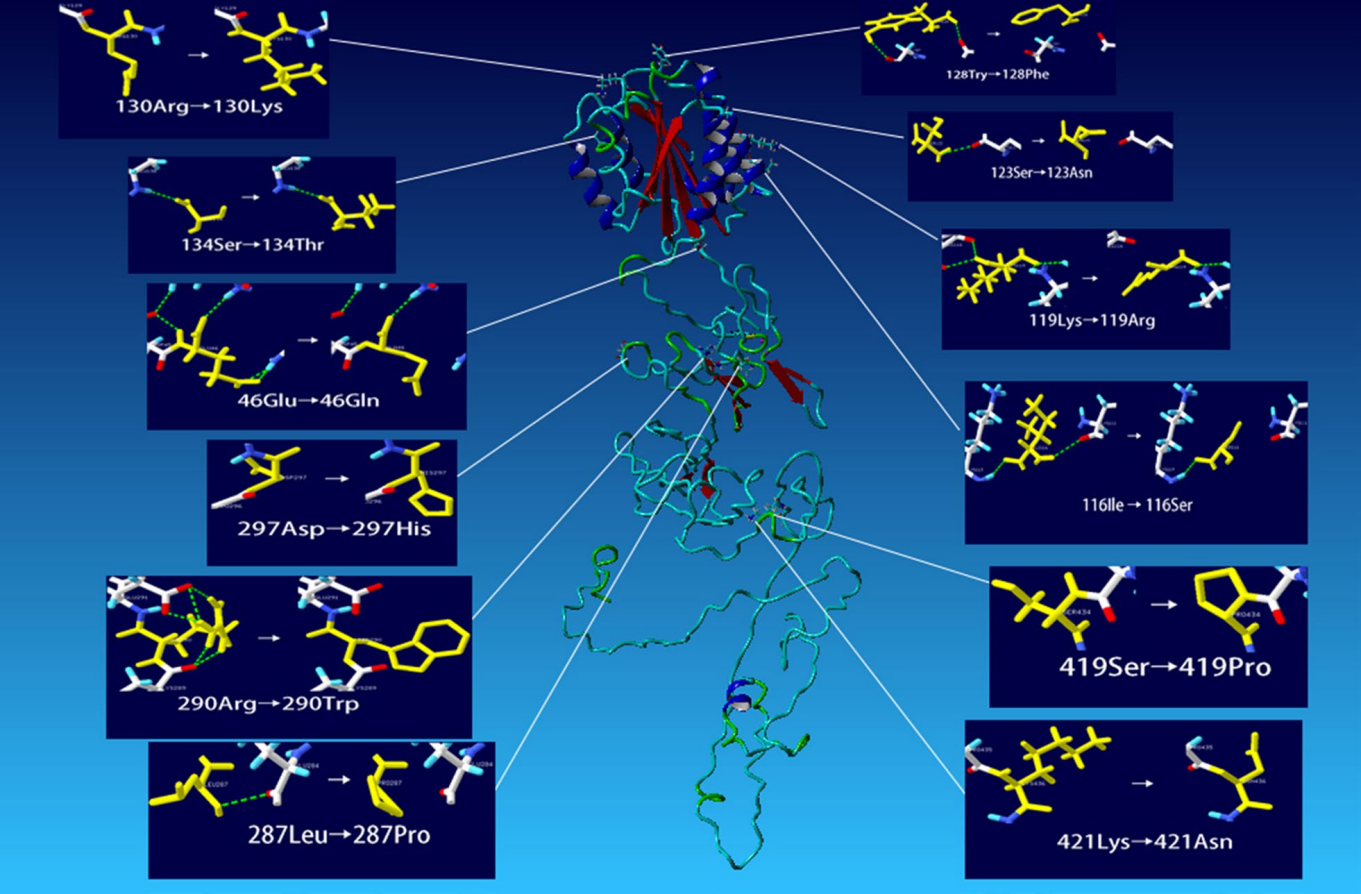
Structure changes before and after relatively common (> 10% globally) mutations which located in the T cell epitopes. The primary structures of the target amino acids were marked as yellow. The green dotted line indicated hydrogen bonding

Furthermore, the three dimensions (3D) structure of full-length 3D7 *Pf*TRAP was predicted and modeled by using I-TASSER server and displayed in YASARA application (Fig. 4). Based on the predicted structure of TRAP, the changes in free energy difference before and after the mutations (ΔΔG) were calculated, and the result shows that these mutations L49V, R285G, R285S, P299S and K421N would lead to the destabilization because of the obviously increased free energy difference (ΔΔG > 1). Furthermore, the spatial conformational changes of the twelve relatively common (> 10% globally) mutations at T cell epitopes were predicted and presented in Fig. 5. According to it, 7 amino acid substitutions (E46Q, I116S, K119R, S123N, Y128F, L287P and R290W) were identified as would lead to the breakage of hydrogen bonding, which would further impair the stability of the protein structure after these mutations (Fig. 5).

## Discussion

As *Pf*TRAP was the potential candidate for anti-malarial vaccine and there are several *Pf*TRAP-based vaccines undergoing the clinical trials [25–27], the worldwide information of its polymorphism was necessary and important for design and improvement of an effective vaccine. In this study, *Pf*TRAP gene data of Bioko Island, Equatorial Guinea was presented and submitted to public database, which had improved the global malarial database. Overall, with the polymorphism analysis, Bioko *Pf*TRAP exhibited the high polymorphism, which is consistent with *Pf*TRAP from other African countries but significantly distinct with the relatively low polymorphic Asian *Pf*TRAP. These phenomena consistence with the previous report about *P. falciparum* circumsporozoite protein (*Pf*CSP) gene [28], which also indicated that the similar polymorphic pattern between Bioko and Africa mainland countries. Moreover, the exploration and analysis of other vaccine candidate genes were also analysed previously, including pre-erythrocytic stage CSP gene, erythrocytic phase merozoite surface protein (MSP-1/2) and asexual blood stage apical membrane antigen-1 (AMA-1) gene [28–30]. These results shown that the polymorphism of candidate genes associated with malaria vaccines in Africa has been at a high level for a long time.

As for the natural selection analysis of Bioko Island *Pf*TRAP, the results obtained by the two different analytical methods are not consistent. The result of dN–dS is 6.2231 (p < 0.05), which is significantly hinted at a natural selection, while the value of Tajima’s D is − 0.41438 (p > 0.05), which is not significant based on Tajima’s D significance levels. For these two results are different, it cannot be concluded with certainty that the PfTRAP on Bioko island has been affected by natural selection, but continued monitoring is still recommended.

In the sight of the haplotype distribution and relation network, haplotypes of Bioko and other African countries distributed scattered while Asian ones are tended to cluster. This phenomenon is in line with the polymorphism result, which shows more mutations appear in African area and thereby lead to the abundant haplotypes. Moreover, the vast majority of these haplotypes are presented as singletons and the high prevalence of singleton is probably associated with the intensity of transmission or rapid expansion of population. Even so, the genetic differentiation between African countries shows limited and so do Asian countries, but an obvious genetic differentiation is found between countries which come from different continents. It might be explained with the population segregation of *Anopheles*, the host of *P. falciparum*, caused by the geographical separation. But interestingly, though Bioko Island is an island separated from African mainland by the Atlantic Ocean, its *Pf*TRAP gene shows low genetic differentiation with other African mainland countries. This result might be explained by the work of Guerra et al. [31, 32], which reported that the strong connection of human movement between Bioko and the mainland Equatorial Guinea (EG), determine a high vulnerability of Bioko to malaria importation; these studies reported that the odds of malaria infection in travellers who had been to mainland EG were more than three times the rest of the population, which confirmed that the majority malaria cases are actively imported by off-island travelers to mainland EG [31, 32]. In general, the non-negligible geographical characteristic might provide a new insight for the development of universal *Pf*TRAP-based vaccine.

Antigen polymorphism has been a major obstacle in the way of developing effective vaccines. Recent studies have highlighted the importance of protective roles of CD8+ T-cell and memory T-cell responses to *Pf*TRAP from clinical malaria cases [33, 34]. Mutations on the surface of the antigen make it more difficult for the host immune system to recognize the antigen, and even lead to immune avoidance and reduce the immune effect. The present study had found a large number of substitutions located at the antigen epitopes, and the destructive prediction was presented. The destructive prediction result in this study reflect the potential destructive effect on the protein structure and further impaction of TRAP function. As it is known that TRAP is a protein that plays an important role in the sporozoite gliding motility, hepatocyte invasion and establishment of infection in the vertebrate host [6–8], thus ‘damaging mutations’ might affect these functions but it would not be a threat to survival, which could explain that why the parasites with 'damaging mutations' still alive and be detected. Furthermore, TRAP as a vaccine candidate gene of great potential, the ‘damaging mutations’ probably affect the effectiveness of the vaccine, for the protein surface structure were changed. Several mutations were predicted as damaging (I116T, L122I, Y128F, G228V and P299S), and Y128F is noteworthy with global frequency of 21%. Some ‘possibly damaging’ mutations such as E46Q, I116S and T134S were also found in high frequency globally (24%, 13% and 37%, respectively). As these mutations were in such high frequency and were predicted as damaging to TRAP function or structure, the follow-up continuous monitoring project is worth to pay special attention to.

Nowadays, several malaria vaccines encoding the pre-erythrocytic antigen ME-TRAP, which often coupled with different adjuvants such as Chimpanzee adenovirus (ChAd63) or modified vaccinia Ankara (MVA), had been developed and issued by researchers have entered the clinical trial stage [26, 27]. Still, these effects are not ideal for the goal of developing a globally effective vaccine. Some researchers had put forward a new insight that CSP-TRAP fusion antigens (TRAP N-terminal domains fused to circumsporozoite protein C-terminus with or without repeat region) could produce an effective host immune and it has been validated in vitro cell experiments [35]. In this global analysis, the TRAP N-terminal domains (including Signal Sequences, A-domain and TSR) showed active genetic mutation, especially in A-domain, and there were 8 mutations predicted to do harm to protein structure or function. It presents the suggestion that the application of TRAP N-terminal domains in the vaccine components might deserve more observation and in-depth assessment. Moreover, as the previous research shown, there are numerous epitopes were predicted in the conserved regions that signifies the possibility of the development of a universal TRAP-based malaria vaccine design [36]. As TRAP showed its great potential in inducing immunity, the systematic statistics and analysis of the polymorphism based on the genetic data from global malaria endemic regions shows a more important role in the development of TRAP-related vaccines.

## Conclusion

The overall trend of Bioko *Pf*TRAP and African *Pf*TRAP shows no significant difference. The *Pf*TRAPs from the same continent shows more homogeneity while the ones from different continents shows more heterogeneity. Taken together, results of the present investigation showed that global *Pf*TRAP gene is highly polymorphic (especially at T cell epitopes) and some mutations shows destructive to the protein structure or function, which may lead to a change in the affinity of these epitopes to immune molecules. Moreover, evidences in this analysis supported that the global *Pf*TRAP gene is under natural selection. Although TRAP related vaccine clinical trials have not been deployed on Bioko Island, in this paper, polymorphisms and natural selection of *Pf*TRAP are analysed to provide relevant genetic background information for future deployment of clinical trials.

## Supplementary Information

**Additional file 1.** Global PfTRAP SNP detail information.

**Additional file 2.** Geographical distribution of haplotypes.

**Additional file 3.** Haplotype information.

**Additional file 4.** 1287 global PfTRAP sequences from Pf3K Database.

**Additional file 5.** Original file for haplotype network.

**Additional file 6.** Detail information of CD8+ T cell epitopes of PfTRAP.

## Data Availability

All data generated or analysed during this study are included in this published article [and its additional information files].

## Abbreviations

*Pf*TRAP: *Plasmodium falciparum* Thrombospondin-related adhesive protein
BIMEP: Bioko Island Malaria Elimination Project
BIMCP: Bioko Island Malaria Control Project
EGMVI: Equatorial Guinea Malaria Vaccine Initiative
SNP: Single nucleotide polymorphism
PDCs: Private diagnostic clinics
EIR: Entomological inoculation rate
RDT: Rapid diagnostic test
HRM: High-resolution melting
PCR: Polymerase chain reaction
Rb: Recombination between adjacent sites
Rm: The minimum number of recombination events
MIDAS: Metal ion-dependent adhesion site
FST: Fixation Index
AMA-1: Apical membrane antigen-1
CSP: Circumsporozoite protein
MSP-1/2: Merozoite surface proteins
EG: Equatorial Guinea
