# Supplementary material for: Genetic diversity and natural selection on the thrombospondin-related adhesive protein (TRAP) gene of Plasmodium falciparum on Bioko Island, Equatorial Guinea and global comparative analysis
Source: Malar J. 2021 Mar 2;20:124. doi: 10.1186/s12936-021-03664-8 (PMC7922716; doi:10.1186/s12936-021-03664-8)
Supplement: Supplementary file 6 — Additional file 6. Detail information of CD8+ T cell epitopes of PfTRAP. [file 12936_2021_3664_MOESM6_ESM.docx]

Detail information of CD8+ T cell epitopes of PfTRAP.

| ID | positive MHC molecule | | | | | positive T cell assay | |
| --- | --- | --- | --- | --- | --- | --- | --- |
| 24217 | HLA-A*02:01 |  |  |  |  | cytotoxicity | IFNg release |
| 16652 | HLA-A*02:01 | HLA-A*02:02 | HLA-A*02:03 | HLA-A*02:06 | HLA-A*68:02 | cytotoxicity | IFNg release |
| 42203 |  |  |  |  |  | cytotoxicity |  |
| 14647 |  |  |  |  |  | cytotoxicity |  |
| 4506 | HLA-B8 |  |  |  |  | cytotoxicity |  |
| 7640 |  |  |  |  |  | cytotoxicity |  |
| 28325 |  |  |  |  |  | cytotoxicity |  |
| 32525 | HLA-B8 |  |  |  |  | cytotoxicity |  |
| 32526 | HLA-B8 |  |  |  |  | cytotoxicity | IFNg release |
| 2726 |  |  |  |  |  | cytotoxicity |  |
| 37827 |  |  |  |  |  | cytotoxicity |  |
| 38775 |  |  |  |  |  | cytotoxicity |  |
| 65367 |  |  |  |  |  | cytotoxicity |  |
| 13604 |  |  |  |  |  | cytotoxicity |  |
| 6158 |  |  |  |  |  | cytotoxicity |  |
| 6618 |  |  |  |  |  | cytotoxicity |  |
| 34701 | HLA-A*11:01 | HLA-A*03:01 | HLA-A*33:01 | HLA-A*68:01 | HLA-A*31:01 | cytotoxicity | IFNg release |
| 37073 | HLA-A*03:01 | HLA-A*11:01 | HLA-A*68:01 | Mamu-A1*002:01 |  | cytotoxicity | IFNg release |
| 37074 | HLA-A*03:01 | HLA-A*11:01 | HLA-A*31:01 | HLA-A*33:01 | HLA-A*68:01 | cytotoxicity | IFNg release |
| 2681 |  |  |  |  |  | cytotoxicity |  |
| 65810 | HLA-B*07:02 | HLA-B*35:01 | HLA-B*51:01 | HLA-B*53:01 |  | cytotoxicity | IFNg release |
| 3546 |  |  |  |  |  | cytotoxicity |  |
| 64808 |  |  |  |  |  | cytotoxicity |  |
